# Serum Creatinine Distinguishes Duchenne Muscular Dystrophy from Becker Muscular Dystrophy in Patients Aged ≤3 Years: A Retrospective Study

**DOI:** 10.3389/fneur.2017.00196

**Published:** 2017-05-08

**Authors:** Liang Wang, Menglong Chen, Ruojie He, Yiming Sun, Juan Yang, Lulu Xiao, Jiqing Cao, Huili Zhang, Cheng Zhang

**Affiliations:** ^1^Department of Neurology, The First Affiliated Hospital, Sun Yat-sen University, Guangzhou, Guangdong, China; ^2^Department of Health Care, The First Affiliated Hospital, Sun Yat-sen University, Guangzhou, Guangdong, China; ^3^Department of Neurology, Zhujiang Hospital, Southern Medical University, Guangzhou, Guangdong, China; ^4^The Department of Tissue Typing Center, Nanfang Hospital, Southern Medical University, Guangzhou, Guangdong, China; ^5^Department of Neurology, Wuhan Central Hospital, Wuhan, Hubei, China; ^6^Department of Neurology, Guangzhou First People’s Hospital, Guangzhou, Guangdong, China

**Keywords:** Becker muscular dystrophy, Duchenne muscular dystrophy, dystrophinopathy, serum creatinine, molecular markers

## Abstract

Here, we investigated correlations between serum creatinine (SCRN) levels and clinical phenotypes of dystrophinopathy in young patients. Sixty-eight patients with dystrophinopathy at the Neuromuscular Clinic, The First Affiliated Hospital, Sun Yat-sen University, were selected for this study. The diagnosis of dystrophinopathy was based on clinical manifestation, biochemical changes, and molecular analysis. Some patients underwent muscle biopsies; SCRN levels were tested when patients were ≤3 years old, and reading frame changes were analyzed. Each patient was followed up, and motor function and clinical phenotype were assessed when the same patients were ≥4 years old. Our findings indicated that in young patients, lower SCRN levels were associated with increased disease severity (*p* < 0.01) and that SCRN levels were the highest in patients exhibiting mild Becker muscular dystrophy (BMD) (*p* < 0.001) and the lowest in patients with Duchenne muscular dystrophy (DMD) (*p* < 0.01) and were significantly higher in patients carrying in-frame mutations than in patients carrying out-of-frame mutations (*p* < 0.001). SCRN level cutoff values for identifying mild BMD [18 µmol/L; area under the curve (AUC): 0.947; *p* < 0.001] and DMD (17 µmol/L; AUC: 0.837; *p* < 0.001) were established. These results suggest that SCRN might be a valuable biomarker for distinguishing DMD from BMD in patients aged ≤3 years and could assist in the selection of appropriate treatment strategies.

## Introduction

Dystrophinopathy is a recessive X-linked hereditary disease with an incidence of 1 in 3,500 newborn males, characterized by progressive muscle weakness, atrophy, and loss of ambulation (1). The disease can be divided into two clinical phenotypes according to different courses: Duchenne muscular dystrophy (DMD) and Becker muscular dystrophy (BMD). DMD exhibits rapid progression and loss of ambulation prior to age 12, whereas BMD exhibits relatively mild symptoms with slower progression. Following the successful cloning of *DMD* in 1985, the molecular pathogenesis of the disease was determined, and the importance of dystrophin in maintaining normal muscle function was discovered (2, 3). Different *DMD* mutations result in two primary forms of defective dystrophin: a truncated, non-functional protein associated with DMD and a partially functional protein associated with BMD (4).

Current treatment regimens for dystrophinopathy can delay the age at which loss of ambulation occurs and improve the patient’s life span, which are insufficient (5). As technology improves, new prospective treatments are proposed, such as stem cell therapy and gene therapy, including exon skipping and stop codon readthrough therapy (6–8). However, issues such as side effects and high costs remain unresolved (9). Therefore, they are not recommended for patients with mild BMD. As early treatment initiation leads to improved outcomes, identifying specific clinical phenotypes in young patients is essential for selecting appropriate treatments (10).

Prior to loss of ambulation, three main methods are used to distinguish clinical phenotypes: genotype–phenotype correlation, evaluation of dystrophin levels in the muscle tissues, and clinical manifestation. Genotype–phenotype correlations comprise the majority of the cases, with still some exceptions in both the dystrophies, but more commonly in cases with BMD (11, 12). Besides, genetic testing is expensive and time consuming, limiting its application in biomarker assessment. In addition, dystrophin expression does not always constitute an accurate marker of clinical phenotypes (13–15). Clinical manifestation is helpful to distinguish when patients exhibit obvious symptoms, which is therefore less useful in young asymptomatic or paucisymptomatic patients. While creatine kinase expression generally differs between patients with BMD and DMD, a high degree of interpatient and intrapatient variability makes accurate diagnosis difficult (16). Magnetic resonance imaging of the muscle tissue is another prospective method to distinguish the clinical phenotype; however, younger patients likely require sedation to ensure accurate results (17). Therefore, new biomarkers are needed to aid in distinguishing clinical phenotypes in young patients.

Recent studies reported correlations between serum creatinine (SCRN) and dystrophinopathy phenotypes, with differences in the creatine metabolism pathway between patients with DMD and controls particularly striking (16, 18). SCRN, primarily produced in the muscle tissues, plays an important role in energy metabolism, and dysfunctional creatine metabolism has been reported in dystrophinopathy (19, 20). However, the correlation between SCRN levels and the clinical phenotypes in young patients aged ≤3 years with indistinguishable symptoms remains unknown. Therefore, this study investigated the relationships between SCRN levels and clinical dystrophinopathy phenotypes to determine the effectiveness of SCRN as a biomarker with applications in treatment strategy decision-making.

## Materials and Methods

### Study Participants

Sixty-eight patients with dystrophinopathy, admitted to the Neuromuscular Clinic at The First Affiliated Hospital, Sun Yat-sen University for regular visits, participated in this study. The diagnosis was confirmed by clinical manifestation, biochemical changes, and molecular analysis. A proportion of patients underwent a muscle biopsy, some of whom participated in our previous study (18, 21). All participants were male, and none was undergoing glucocorticoid treatment. Participants had a minimum age at their last checkup of 4.06 years, a maximum age of 11.04 years, and a median age of 4.92 years (interquartile range: 4.38–6.23 years). All participants were old enough to be adequately assessed. Clinical phenotypes were divided into mild BMD, severe BMD, and DMD according to the clinical manifestation. Patients with mild BMD exhibited completely normal gross motor functions, those with severe BMD exhibited higher degree of motor dysfunctions, and those with DMD exhibited severe motor dysfunctions. The typing criteria were adjusted according to age. For example, an 11-year-old patient who was unable to run or jump high, but who could stand from a squatting position or climb stairs slowly without using the railing, was classified as severe BMD, as this manifestation was milder than that observed in an 11-year-old patient with DMD.

Patients exhibiting the following confounding factors were excluded from the study: (a) increased SCRN levels owing to renal injury, diabetes mellitus, or consumption of certain drugs (22); (b) decreased SCRN levels owing to obvious malnutrition (determined either by clinical manifestation or decreased serum albumin levels), vegetarian diet, or advanced liver disease (23, 24).

### Sample Processing

Blood samples were collected when the patients were ≤3 years old, at least 4 h after eating to decrease the influence of meat intake on SCRN levels, and were analyzed at our hospital (25). SCRN levels were then measured immediately. At the time of testing, patients had a minimum age of 4 months and a maximum age of 3.88 years (median: 3.16 years; interquartile range: 2.37–3.49 years).

Five milliliters of blood from each patient were centrifuged at 1,810 *g* for 5 min at 24°C, before sera were injected into individual tubes and analyzed using a Vitros 5.1FS chemistry analyzer (Johnson & Johnson Corporation, New Brunswick, NJ, USA). Concomitantly, 2 mL of blood from each patient was deposited in a separate tube for use in *DMD* mutation analysis. Multiplex ligation-dependent probe-amplification reactions were used to detect large sequence rearrangements, and next-generation sequencing, using an Illumina HiSeq 2000 system (Illumina Corporation, San Diego, CA, USA), was used to detect smaller scale mutations. The average sequencing depth was >200×.

### Evaluation of Motor Function

As all participants exhibited normal upper limb motor functions, only lower limb motor function was evaluated, using the Vignos scale (18). Patients were required to walk, climb stairs under protection of their parents, and rise from chairs. Parents, with children failing to comply with instructions, were asked to provide details concerning conditions at home. Patients capable of walking and climbing stairs without assistance were scored as grade 1; walking and climbing stairs with the aid of the railing were scored as grade 2; walking and climbing stairs with the aid of the railing and requiring >25 s to complete eight standard steps were scored as grade 3; walking unassisted and rising from chairs, but who were unable to climb stairs were scored as grade 4; walking unassisted, but who were unable to rise from chairs or climb stairs were scored as grade 5; walking only with assistance or walking independently with long leg braces were scored as grade 6; walking in long leg braces, but requiring assistance for balance were scored as grade 7; standing in long leg braces, but unable to walk, even with assistance, were scored as grade 8; requiring a wheelchair were scored as grade 9; and confined to bed were scored as grade 10.

### Assessment of Reading Frame Rule

Leiden *DMD* reading frame checker (http://www.dmd.nl/index.html) was used to determine whether patients with large genetic rearrangements due to mutations also carried frameshift mutations in *DMD* (11).

### Statistical Analysis

Serum creatinine analysis included 68 data points. Statistical analysis was performed using SPSS version 20.0 (IBM Corp., Chicago, IL, USA), GraphPad PRISM version 7.01 (GraphPad Software, San Diego, CA, USA), and MedCalc version 16.8 (MedCalc Software, Ostend, Belgium). The distribution of data for each variable was assessed using the Shapiro–Wilk test (*n* ≤ 50), and normally distributed data were presented as mean ± SD, with others presented as medians and the 25th and 75th percentiles [median (interquartile range: P_25_–P_75_)]. In addition, 95% confidence interval was calculated. To analyze differences between normally distributed variables, Student’s *t*-test (two variables) or one-way analysis of variance (three variables) was used. To analyze differences between non-normally distributed variables, Mann–Whitney *U* test (two variables), or Kruskal–Wallis *H* test (three variables) was used, applying Bonferroni corrections to the differences between every potential pair of the three variables.

Receiver operating characteristic (ROC) curves were used to generate cutoff values, and the global performance of this test was assessed by determining the area under the curve (AUC). Differences in cutoff values between mild and non-mild BMD, and between DMD and non-DMD, were then analyzed. Cutoff values resulting in the largest Youden index (sensitivity + specificity − 1) were considered optimal. Considering the population of patients with childhood-onset dystrophinopathy, the prevalence of DMD was 12.57/100,000 boys, while the prevalence of BMD was 1.35/100,000 boys (26). Positive-predictive values (PVs+) and negative-predictive values were estimated according to this dystrophinopathy prevalence. All tests were two tailed. For the Shapiro–Wilk test, *p* < 0.1 was considered statistically significant. For all other tests, *p* < 0.05 was considered statistically significant.

## Results

### Clinical Data

Patient characteristics, including clinical features, were summarized in Table 1. The distribution of mutation types was similar to that reported in previous studies (11, 21, 27). Median ages at the time of SCRN testing of patients with mild BMD, severe BMD, and DMD were 3.27 years (interquartile range: 2.68–3.78 years; 95% confidence interval: 2.74–3.76 years), 3.24 years (interquartile range: 2.93–3.71 years; 95% confidence interval: 3.08–3.60 years), and 3.05 years (interquartile range: 2.00–3.43 years; 95% confidence interval: 2.46–3.30 years), respectively. Differences in the ages of the patients between the different groups were not significant (*p* = 0.082). The median SCRN level for all patients was 17.00 µmol/L (interquartile range: 14.00–20.00 µmol/L; 95% confidence interval: 16.00–18.00 µmol/L).

**Table 1:** **The number and ethnic percentages of patients**.

	Number	%
Ethnic	68	
Mongoloid	68	100.00
Nation	68	
Han	68	100.00
Region	68	
North China	2	2.94
South China	66	97.06
Clinical phenotype	68	
Mild BMD	10	14.71
Severe BMD	18	26.47
DMD	40	58.82
Vignos scale	58	
1	29	50.00
2	28	48.28
3	1	1.72
Mutation analysis	68	
Deletion	46	67.65
Duplication	5	7.35
Point mutation	17	25.00

*BMD, Becker muscular dystrophy; DMD, Duchenne muscular dystrophy*.

### SCRN Levels Differ between Mild BMD, Severe BMD, and DMD

**Figure 1:**
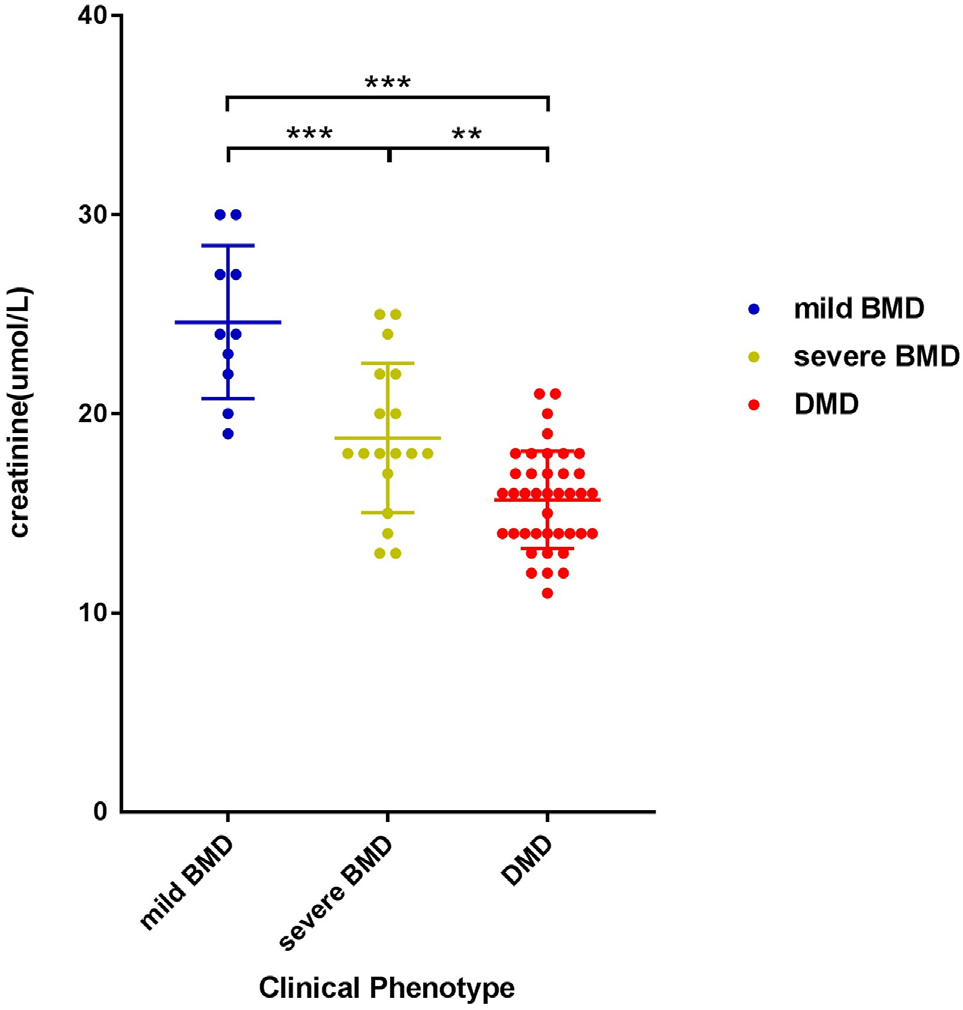
**Scatter plot of serum creatinine levels versus phenotypes for dystrophinopathy patients**. Differently colored dots signify different clinical phenotypes. Differences between different phenotypes were significant (**p < 0.01, ***p < 0.001).

The negative correlation between SCRN level and Vignos score indicated that a more severe disease course corresponded to a lower SCRN level at an early age (*p* < 0.01, data not shown). The SCRN levels of patients at different ages, exhibiting different phenotypes, were analyzed. Among 2-year-old patients at the time of SCRN testing, levels differed significantly between patients with mild BMD, severe BMD, and DMD (*p* = 0.001). SCRN levels in patients with mild BMD (24.25 ± 3.40 µmol/L; 95% confidence interval: 18.83–29.67 µmol/L) were significantly higher than in those with either severe BMD (18.25 ± 3.86 µmol/L*;* 95% confidence interval: 12.10–24.40 µmol/L; *p* = 0.013) or DMD (15.30 ± 2.50 µmol/L; 95% confidence interval: 13.51–17.09 µmol/L; *p* < 0.001), whereas SCRN levels did not differ significantly between patients with severe BMD and DMD (*p* = 0.354). Among 3-year-old patients at the time of testing, SCRN levels also differed significantly between the three clinical phenotypes (*p* < 0.05). Analysis of SCRN levels in patients at different ages who exhibited the various clinical phenotypes indicated that SCRN levels did not differ significantly at different ages (*p* > 0.05). After merging data for similar clinical phenotypes at different ages, patients exhibiting mild BMD symptoms displayed the highest SCRN levels, while those with DMD displayed the lowest (*p* < 0.01; Figure 1), suggesting that higher SCRN levels at an early age correlated with a milder phenotype.

### Correlations between SCRN and Genotype

**Figure 2:**
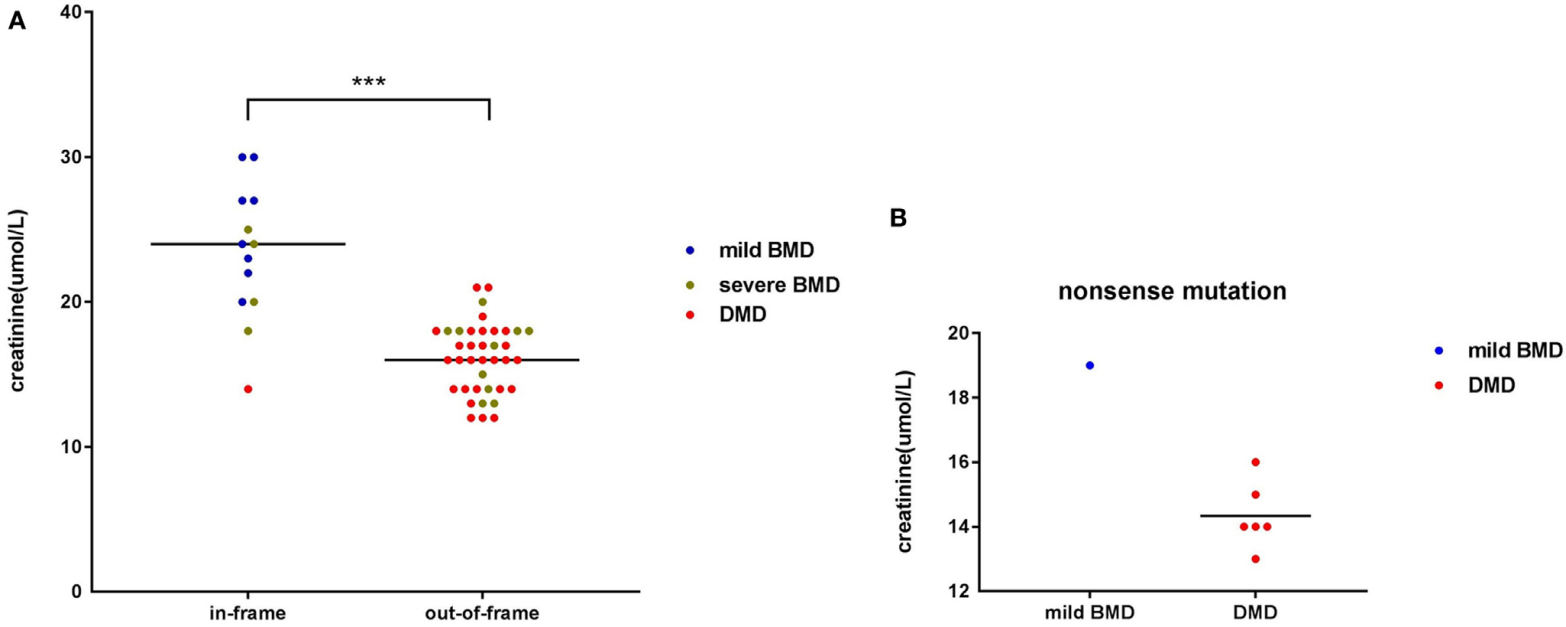
**Scatter plot of serum creatinine (SCRN) levels versus different genotypes for dystrophinopathy patients**. **(A)** Differently colored dots signify different clinical phenotypes. Differences in SCRN levels between groups exhibiting different changes to open reading frames were significant. **(B)** Differently colored dots signify different clinical phenotypes (***p < 0.001).

Among participants with large genetic rearrangements, 13 patients (25.49%) with in-frame mutations and 38 patients (74.51%) with out-of-frame mutations were found. Ages at the time of SCRN testing did not differ significantly between these two groups [3.12 years (interquartile range: 2.85–3.74 years; 95% confidence interval: 2.87–3.71 years) versus 3.17 years (interquartile range: 2.11–3.41 years; 95% confidence interval: 2.53–3.31 years), for patients with in-frame and out-of-frame mutations, respectively; *p* = 0.210]. However, SCRN levels were significantly higher in the in-frame group [23.38 ± 4.65 µmol/L (95% confidence interval: 20.58–26.19 µmol/L)] than in the out-of-frame group [16.00 µmol/L (interquartile range: 14.00–18.00 µmol/L; 95% confidence interval: 15.50–17.00 µmol/L); *p* < 0.001; Figure 2A]. SCRN data for seven patients carrying nonsense mutations are shown in Figure 2B. These data indicated that SCRN levels abide by the reading frame rule and that SCRN levels in exceptional cases might be consistent with phenotypes.

### Determination of Cutoff Values

**Figure 3:**
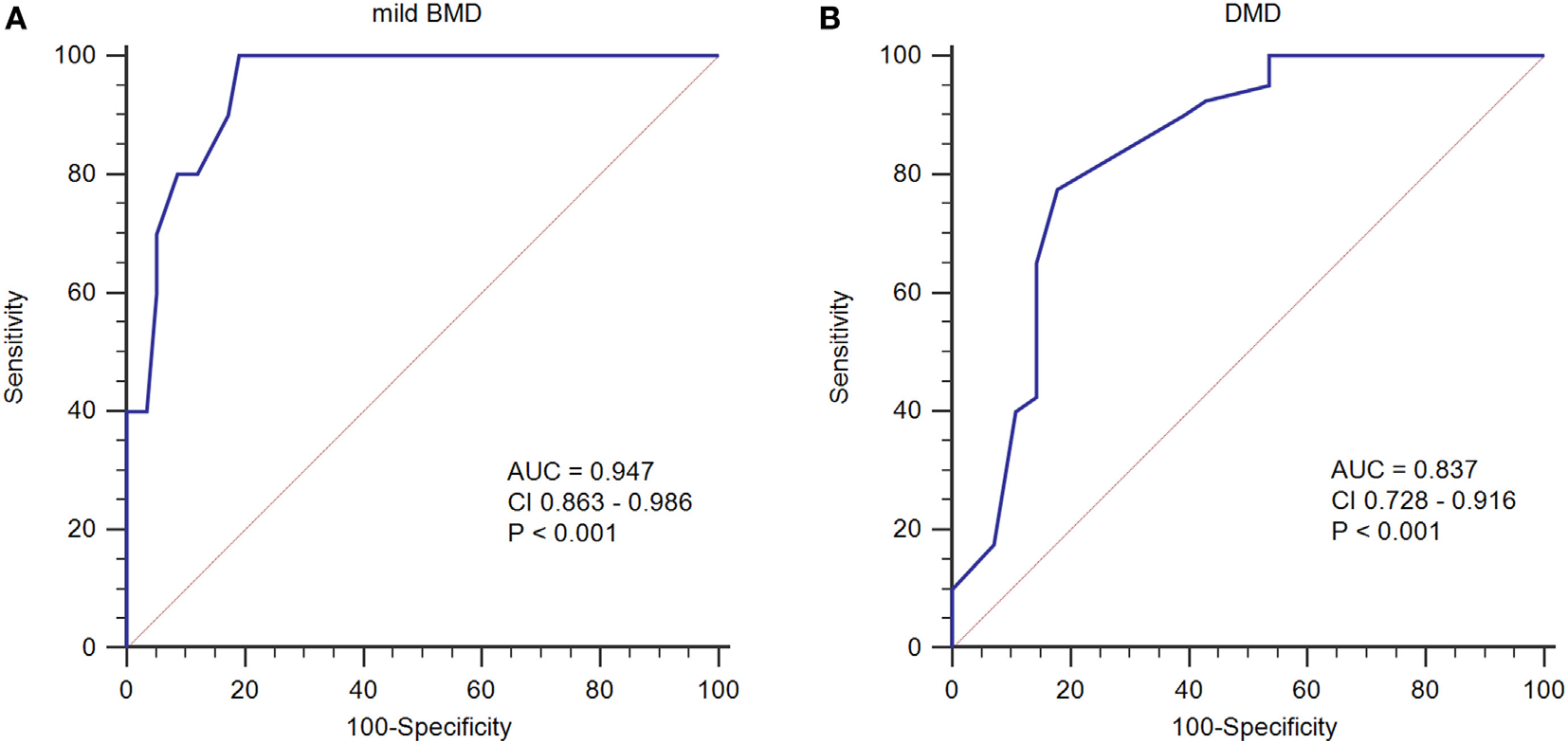
**Receiver operating characteristic curve plots for serum creatinine (SCRN) levels**. **(A)** SCRN levels for identifying patients with mild forms of Becker muscular dystrophy (BMD). **(B)** SCRN levels for identifying patients with Duchenne muscular dystrophy (DMD).

To determine an SCRN level cutoff that could be used to distinguish mild BMD, ROC curve analysis was performed (Figure 3A). The AUC was 0.947 (*p* < 0.001), and the SCRN level cutoff value giving the largest Youden index (81.03%) was 18 µmol/L; with PV+ of <36.2% as the prevalence of BMD, which includes both mild and severe cases, was used to estimate the prevalence of mild BMD. Currently, there are no accurate data concerning the prevalence of mild BMD specifically.

A similar approach was used to determine an SCRN level cutoff that could be used to distinguish DMD (Figure 3B). In this case, the AUC was 0.837 (*p* < 0.001), and the SCRN level cutoff value giving the largest Youden index (59.64%) was 17 µmol/L. Taken together, these results provided SCRN level cutoff values that could be used to distinguish between cases of mild BMD and DMD. Detailed information was provided in Table 2.

**Table 2:** **Cutoff values and potential applications for exon skipping therapy**.

Cutoff value (μmol/L)	Sensitivity (%)	Specificity (%)	Youden index (%)	PV+ (%)	PV− (%)	Condition	Exon skipping
≥25 for mild BMD	40	100	40	100	93.9	Mild BMD	No
≥18 for mild BMD	100	81.03	81.03	≤36.2	100	If <18, not mild BMD	If <18, should consider
≤17 for DMD	77.5	82.14	59.64	97.6	28.2	Probably DMD	Recommended
≤12 for DMD	10	100	10	100	10.7	DMD	Strongly recommended

*BMD, Becker muscular dystrophy; DMD, Duchenne muscular dystrophy; PV+, positive-predictive value; PV−, negative-predictive value*.

## Discussion

This study, based on previously reported results, investigated correlations between SCRN levels and dystrophinopathy (18). Sixty-eight patients, who were first sampled when they were young children with no obvious motor dysfunction, went on to develop some motor dysfunction by their last checkup. Our results indicated that SCRN level was associated with disease severity, clinical phenotype, and genotype.

Our present findings suggest that patients with lower SCRN levels at young ages go on to develop more severe motor dysfunction and more severe disease phenotype as they age, indicating that SCRN level at a younger age is predictive of disease progression. Herein, we propose the following hypothesis to explain these observations. SCRN is associated with energy metabolism within muscle, and its levels are proportional to creatine and phosphocreatine degradation within muscle tissue (19). SCRN levels have been used to estimate muscle mass (28, 29); indeed, muscular dystrophy-associated reductions in muscle mass and creatine metabolism dysfunction are known to affect SCRN levels (20, 30, 31). Thus, SCRN levels in pediatric patients with dystrophinopathy are lower than in healthy children (16, 18). Patients with dystrophinopathy exhibit muscular lesions from the fetal stage, implying that the lesions in patients with slowly progressing BMD reflect a more moderate histopathology relative to that seen in patients with DMD (32); patients with DMD possess more severe muscular lesions than patients with BMD, even if they are asymptomatic during early childhood, resulting in reduced muscle mass and more severe creatine metabolism dysfunction as DMD becomes apparent. This hypothesis might explain the observed differences in SCRN levels between different clinical phenotypes.

Previously, we proposed a correlation between SCRN level and genotype (18). In this study, we demonstrated that SCRN levels might reflect the type of genetic mutation found in patients with dystrophinopathy. Particularly, SCRN levels of two cases with exceptional genotype–phenotype correlations, a DMD case with in-frame mutation and mild BMD case with a nonsense mutation, were consisted with clinical phenotype. While out-of-frame and nonsense mutations are thought to result in relatively severe phenotypes associated with DMD, there are some exceptions where special mechanisms, such as alternative splicing, occur (33–35). In the two exceptional cases noted above, genotype modifications may have occurred by unusual mechanisms, and the observed SCRN levels may be indicative of phenotypic differences related to these modifications. Therefore, it is possible that SCRN levels supplement the deficiencies of genotyping in predicting clinical phenotypes and act as a useful biomarker. Given that SCRN levels can be analyzed quickly, easily, and cost effectively in virtually all hospitals, SCRN measurements could be taken before deciding on a treatment strategy without significantly delaying treatment. The SCRN level cutoff values that we report here as being indicative of specific disease phenotypes could be useful in practical clinical applications, aiding clinicians when choosing treatment strategies. Table 2 shows cutoff values that could be used prior to the initiation of exon skipping treatment.

There were some limitations to this study that should be considered when interpreting the data. First, despite excluding confounding factors that could influence SCRN levels, such as age, ethnicity, diet, and renal injury, other potential confounders including fluid status could not be excluded (25). In the minority of older patients, some large shifts in SCRN level were observed (data not shown) for reasons that remain unclear. Second, the SCRN level distribution curves for the different clinical phenotypes contained some overlapping, making it difficult to discern clinical differences using SCRN levels exclusively. Third, our study included few patients aged <2 years, because these patients are generally almost asymptomatic and so not easily identifiable. However, we believe that the results of this study can be still applied to the whole patient population (age ≤3) because the SCRN level distribution was similar across patients of different ages. Finally, the follow-up duration was not ideal, and longer observational periods are necessary to make definitive conclusions.

In summary, SCRN levels in patients with dystrophinopathy aged ≤3 years constitute a potential biomarker for distinguishing clinical phenotypes that overcomes some of the disadvantages associated with existing methods. Furthermore, the use of SCRN as a biomarker can potentially aid clinicians when determining treatment strategies for patients with dystrophinopathy.

## Ethics Statement

This study was carried out in accordance with the recommendations of “guidelines for clinical study, ICE for Clinical Research and Animal Trials of the First Affiliated Hospital of Sun Yat-sen University.” The protocol was approved by the “ICE for Clinical Research and Animal Trials of the First Affiliated Hospital of Sun Yat-sen University.” This study was approved to waive the informed parental consents by “ICE for Clinical Research and Animal Trials of the First Affiliated Hospital of Sun Yat-sen University,” because it was impossible to get written informed parental consents from every participant, and this study did not present personal information and was not harmful to any participant.

## Author Contributions

WL and CM designed the study, analyzed the data, and drafted the manuscript. HR, SY, YJ, CJ, and ZH assisted in clinical data collection. XL and ZC assisted in data analysis and in drafting the manuscript. WL and CM contributed equally.

## Conflict of Interest Statement

The authors declare that the research was conducted in the absence of any commercial or financial relationships that could be construed as a potential conflict of interest. The reviewer, RJ, and handling editor declared their shared affiliation, and the handling editor states that the process nevertheless met the standards of a fair and objective review.

## Abbreviations

SCRN, serum creatinine; DMD, Duchenne muscular dystrophy; BMD, Becker muscular dystrophy; AUC, area under the curve; ROCs, receiver operating characteristics; PV+, positive-predictive value; PV−, negative-predictive value.
